# Proactive Control in Bilingual Language Switching: Evidence from Cue-Based Preparation in Croatian-German Children

**DOI:** 10.3390/bs16091624

**Published:** 2026-09-11

**Authors:** Emily Lončarek, Maki Kubota

**Affiliations:** Department of Linguistic, Literary and Aesthetic Studies, University of Bergen, 5007 Bergen, Norway; emilylon4@gmail.com

**Keywords:** bilingual language control, proactive control, language switching, children, preparation time, cognitive development

## Abstract

This study investigates a previously unexplored aspect of bilingual language control in young children: the ability to engage proactive control mechanisms during language switching. Using a novel cued language-switching paradigm, we examined whether Croatian-German bilingual children aged 4–11 can utilize advance preparation time to facilitate language switching, and whether this ability varies with age. Thirty-nine bilingual children completed a picture-naming task in which a language cue (flag) either appeared simultaneously with the target picture (no preparation) or preceded it by a brief interval (preparation possible). Results revealed a significant cue benefit, with children responding faster when given advance preparation time. This benefit was present across the sampled age range and was observed in every child who completed both cue conditions, suggesting that even the youngest children in our sample (4–6 years; n = 7) were able to use anticipatory information to prepare their responses. Additionally, we observed reliable switch costs (switch > stay) that did not differ detectably by target language or language dominance, alongside mixing costs that were modulated by group, a pattern partly attributable to baseline naming differences. These findings provide preliminary evidence that anticipatory preparation processes in bilingual language switching may emerge early in development. The results have important implications for understanding the interplay between bilingual experience, cognitive development, and the emergence of anticipatory control mechanisms in childhood.

## 1. Introduction

Bilingual individuals face a unique cognitive challenge: managing two linguistic systems that are simultaneously active yet must be selectively deployed (Green, 1998; Kroll & Bialystok, 2013). This challenge is particularly evident during language switching, where bilinguals must shift from producing words in one language to another. A substantial body of research has documented the cognitive costs associated with such switching, including switch costs, i.e., the performance decrements observed when switching languages compared to staying in the same language, and mixing costs, the sustained costs of maintaining dual-language readiness even on non-switch trials (Declerck et al., 2015; Meuter & Allport, 1999). However, a critical yet underexplored question concerns the role of anticipatory preparation in facilitating language switching, especially in young bilingual children.

The distinction between reactive and proactive control has become increasingly central to understanding cognitive control more broadly (Braver, 2012). Reactive control refers to the “just-in-time” mobilization of control processes in response to conflict or interference, while proactive control involves the anticipatory, sustained activation of goal-relevant information to optimize performance before conflict arises. In the domain of language switching, reactive control would involve suppressing the non-target language only after detecting interference, whereas proactive control would entail preparing the target language in advance based on predictive cues. While extensive research has documented reactive control processes in bilingual language switching through the study of switch and mixing costs, the capacity for proactive control in this domain (particularly in children) remains largely unexplored.

This gap is particularly striking given the developmental literature suggesting that proactive control mechanisms emerge relatively late in childhood, with children under 7–8 years showing a predominant reliance on reactive strategies (Chatham et al., 2009; Munakata et al., 2012). If this developmental trajectory applies to bilingual language control, young bilingual children should show limited ability to benefit from advance preparation cues during language switching. Alternatively, within the specific domain of language switching, bilingual children accumulate extensive everyday practice at selecting a language from contextual cues, and this domain-specific experience might allow them to exploit an advance language cue earlier than general accounts of proactive-control development would predict—even if their proactive control in non-linguistic switching tasks follows the usual, later developmental trajectory.

The present study addresses this gap by introducing a novel manipulation of preparation time in a bilingual language-switching task with Croatian–German bilingual children aged 4 to 11 years. Critically, this is, to our knowledge, one of the first studies to systematically examine whether young bilingual children can utilize advance language cues to engage proactive control during language switching, and whether this ability shows developmental changes across early and middle childhood. We compare two conditions: one in which the language cue (a flag indicating Croatian or German) appears simultaneously with the picture to be named (no preparation), and another in which the cue precedes the picture by a brief interval (preparation condition). This manipulation allows us to directly assess children’s capacity for anticipatory language selection.

Beyond examining proactive control, this study also investigates how language dominance shapes both transient and sustained control demands in bilingual language switching. We compare Croatian-dominant children living in Croatia with German-dominant children living in Austria, groups that differ markedly in their daily language exposure and switching contexts. This design allows us to examine whether the patterns of switch costs and mixing costs (and their relationship to preparation) vary as a function of children’s dominant language and linguistic environment.

### 1.1. Reactive and Proactive Control

The Dual Mechanisms of Control (DMC) framework (Braver, 2012) distinguishes between two fundamentally different modes of cognitive control that differ in their temporal dynamics, resource demands, and functional characteristics. Reactive control operates in a transient, stimulus-driven manner, mobilizing control processes only when needed to resolve conflict or interference as it arises. This “late correction” mechanism is characterized by relatively low sustained costs but potentially higher interference when conflict occurs, as control must be engaged in the moment to handle unexpected demands. In contrast, proactive control operates in an anticipatory, goal-driven manner, actively maintaining task-relevant information in a sustained fashion before conflict occurs. This “early selection” mechanism carries sustained costs, requiring continuous engagement of cognitive resources and related metabolic activity, but can prevent interference before it occurs by preparing the cognitive system in advance.

Within the DMC framework, proactive control comprises several separable components: detecting a predictive cue, decoding its meaning, preactivating goal-relevant representations, maintaining them across a delay, and using them to bias subsequent processing (Braver, 2012). We operationalize proactive language control in this study as the capacity to use an informationally specific language cue, presented in advance of the target, to preactivate the lexical–phonological system of the target language before picture onset. Developmental research has documented a robust shift from predominantly reactive to increasingly proactive control across childhood, with this transition occurring most prominently between ages 5 and 7 years (Chatham et al., 2009; Chevalier et al., 2015; Lucenet & Blaye, 2014; Munakata et al., 2012). This developmental trajectory has been demonstrated across multiple cognitive domains and experimental paradigms. For instance, in the AX-Continuous Performance Test, which requires children to respond to specific prime-probe combinations (e.g., responding when a dog is followed by a cat), preschoolers engage mental effort relatively late by reactively retrieving prime information after the probe appears (“Did I see a dog before the cat?”). By contrast, older children (6 years and above) engage mental effort earlier, right after the prime, by maintaining it in working memory and anticipating the associated probe (“There is a dog now, a cat will probably follow”; Chatham et al., 2009).

This age-related transition from reactive to proactive control reflects not just quantitative improvements in cognitive resources, but qualitative changes in how children approach cognitive challenges. With age and experience, children become increasingly adept at evaluating task demands, assessing their own cognitive resources, and selecting the most appropriate control mode for each situation. Importantly, proactive control does not simply replace reactive control with development; rather, both strategies remain available, and older children and adults flexibly engage either mode depending on task demands (Braver et al., 2009; Chevalier, 2015).

### 1.2. Bilingual Language Control: Inhibition and Switching Costs

The Inhibitory Control Model (Green, 1998) proposes that bilingual language control relies on suppressing the non-target language through inhibitory mechanisms. When bilinguals switch from Language A to Language B, they must overcome the inhibition previously applied to Language B while simultaneously inhibiting Language A. A key prediction is asymmetrical switch costs: returning to the more dominant language should incur greater costs because stronger inhibition is required to suppress it when using the weaker language. For instance, Meuter and Allport (1999) found that unbalanced bilinguals showed larger switch costs when returning to their dominant language (L1) than when switching to their weaker language (L2). Similar asymmetrical patterns have been documented across various language pairs (Costa et al., 2006; Philipp et al., 2007), supporting the view that more proficient languages require stronger suppression and thus greater effort to reactivate. Although this evidence from adult studies supports asymmetrical switch costs, findings across studies remain inconsistent and far from universal. A systematic review by Declerck and Koch (2023) highlights the difficulty of drawing firm conclusions from the existing literature, and large-scale and meta-analytic work has similarly pointed to the role of task-specific demands, individual participant profiles, and contextual variables in determining whether asymmetries emerge at all (Gade et al., 2021; Goldrick & Gollan, 2023).

Research with bilingual children has yielded even more variable results. While some studies report asymmetrical switch costs consistent with the adult pattern (Gross & Kaushanskaya, 2015), others find symmetrical costs where switching difficulty is similar regardless of target language (Declerck et al., 2017). These inconsistencies may reflect several developmental factors. First, inhibitory control mechanisms are still maturing throughout childhood, with substantial development continuing into adolescence (Best & Miller, 2010). Second, language dominance in childhood is often more fluid and context-dependent than in adulthood, varying across home, school, and peer settings (Paradis, 2023). This variability may reduce the systematic inhibitory differences between languages that produce asymmetrical costs in adults with stable dominance profiles. Third, task design factors (such as cue type, response-to-stimulus intervals, and block structure) can influence whether asymmetries emerge, particularly in child populations (Mosca & Clahsen, 2016).

Beyond transient switch costs, research has identified mixing costs (also called global costs), which is the sustained performance decrements observed on non-switch trials in mixed-language blocks compared to single-language blocks. While switch costs reflect the momentary demands of reconfiguring from one language to another, mixing costs reflect the sustained demands of maintaining dual-language readiness and monitoring for potential switches (Los, 1996; Rubin & Meiran, 2005). Children show reliable mixing costs in language-switching tasks, and these costs appear particularly sensitive to language experience and proficiency (Kubota et al., 2020; Gross & Kaushanskaya, 2018). The “weaker links” hypothesis (Gollan et al., 2005) proposes that bilinguals have weaker connections between concepts and words than monolinguals due to reduced practice in each language. For children, who have accumulated less total language experience than adults, these connections may be especially vulnerable to variations in recent exposure and use. Thus, mixing costs in children may reflect both the sustained monitoring demands of dual-language contexts and the language-specific difficulty of maintaining adequate activation of form-meaning mappings, particularly in the weaker language.

Despite extensive research on reactive control processes in language switching, the role of preparation time as a modulator of proactive control has received comparatively limited attention. The cue-to-stimulus interval (CSI) (i.e., the time between the appearance of a language cue and the onset of the target stimulus) provides a direct window into bilingual proactive control by manipulating how much time speakers have to prepare for an upcoming language response.

In adult bilinguals, longer CSIs have been shown to reduce switching costs (Costa & Santesteban, 2004; Fink & Goldrick, 2015; Ma et al., 2016; Mosca & Clahsen, 2016; Verhoef et al., 2009), and under sufficiently long preparation intervals, switching costs can be eliminated entirely (Mosca & Clahsen, 2016; Mosca et al., 2022). This parallels preparation effects long documented in the task-switching literature, where advance cues allow reconfiguration of the upcoming task set (Meiran, 1996). Recent work further suggests that preparation effects are themselves dynamic: with very long intervals, anticipatory activation may dissipate over time (Liu & Chaouch-Orozco, 2025). Preparation time has also been shown to reduce mixing costs, suggesting that it allows bilinguals to recover from the sustained proactive control exerted across mixed-language blocks (Ma et al., 2016). Together, these findings point to preparation time as serving two complementary functions: facilitating dissipation of reactive control applied on the previous trial and allowing relaxation of proactive inhibition on the non-target language. However, no published studies have examined whether young bilingual children can utilize such preparation opportunities. This gap is particularly important given the developmental literature suggesting that proactive control emerges gradually across childhood. If young children lack mature proactive control mechanisms, they should show limited benefit from advance language cues.

### 1.3. Research Questions

This study addresses two primary research questions:

1. Can young bilingual children utilize advance preparation time to facilitate language switching, and does this capacity vary with age?

Based on general developmental models of cognitive control (Chatham et al., 2009; Munakata et al., 2012), we might predict that younger children (under 6–7 years) would show minimal benefit from preparation time, as they rely primarily on reactive control strategies. We test this by comparing performance in preparation versus no-preparation conditions across a broad age range.

2. How do switch costs and mixing costs manifest in bilingual children, and are they modulated by language dominance?

Following the Inhibitory Control Model (Green, 1998), we predict asymmetrical switch costs, with larger costs when returning to the dominant language. However, given mixed findings in child populations, an alternative possibility is that children show symmetrical switch costs, reflecting more general reconfiguration demands rather than dominance-specific inhibition. For mixing costs, we predict modulation by language dominance and target language, with children showing larger costs when maintaining their weaker language in a state of readiness.

## 2. Method

### 2.1. Participants

Thirty-nine Croatian-German bilingual children (19 female, 20 male; age range: 4–11 years, M = 8.5, SD = 2.0; 4 year-olds (N = 2), 5 year-olds (N = 2); 6 year-olds (N = 3); 7 year olds (N = 5); 8 year-olds (N = 6); 9 year-olds (N = 4); 10 year-olds (N = 13); 11 year-olds (N = 4)) participated in the study. Children were recruited from two populations: Croatian-dominant children living in Croatia (n = 20) and German-dominant children living in Austria (n = 19). All children had regular exposure to both languages through family, education, or community contexts but varied in their relative proficiency and daily usage patterns.

The Croatian-dominant group consisted of children who lived in Croatia and used Croatian as their primary home and community language. They had exposure to German primarily through learning German in a school setting. Parental questionnaire data showed that Croatian was the dominant language for daily communication, media consumption, and peer interaction. The German-dominant group consisted of children who lived in Austria and used German as their primary home and community language. They had exposure to Croatian primarily through bilingual parents, heritage language maintenance programs, or visits to Croatia. Table 1 below summarizes the questionnaire data between the two groups of participants.

Language exposure and proficiency were assessed via a parental questionnaire. Exposure items asked what proportion of the time the child spoke and heard each language in a given context (home, school, family, friends, teachers, and media), scored from 0 to 1; the exposure values in Table 1 are means of these proportions per context. Proficiency was assessed by three parental ratings per language (understanding, speaking, and reading) on 5-point scales, summed into a composite score ranging from 3 to 15 per language; the proficiency values in Table 1 refer to this composite. Dominance classification was based on the child’s country of residence and primary home and community language as reported by parents, and was corroborated by the exposure and proficiency measures in Table 1.

The study was approved by the Norwegian Agency for Shared Services in Education and Research (Sikt approval number 840265), and informed consent was obtained from all parents or legal guardians before participation.

### 2.2. Materials and Design

The experiment employed a picture-naming task using 34 common objects drawn from the MultiPic database (Duñabeitia et al., 2018). Items were selected to be familiar to young children and to have translation equivalents in Croatian and German that were unambiguous and age-appropriate (e.g., Ball/Lopta for “ball”; Hund/Pas for “dog”).

Because Croatian and German differ in phonological structure, we compared the 34 translation pairs post hoc on four phonological dimensions. The two item sets did not differ significantly in phonological syllable count (German: M = 1.88, SD = 0.69; Croatian: M = 1.97, SD = 0.76; paired t(33) = −0.62, *p* = 0.540), word-initial onset complexity (number of onset consonants; German: M = 0.97, SD = 0.58; Croatian: M = 1.09, SD = 0.62; t(33) = −0.85, *p* = 0.402), or word length in phonemes (German: M = 4.50, SD = 1.38; Croatian: M = 4.76, SD = 1.48; t(33) = −1.07, *p* = 0.292). Word-final consonant clusters were infrequent in both sets (German: 5 items; Croatian: 2 items; McNemar test, *p* = 0.450). The two item sets were therefore reasonably well matched on gross phonological complexity, although they were not matched pair-by-pair on segmental content, and subtler cross-linguistic differences in segmental realization remain. Importantly, the critical preparation-benefit comparison is a within-language, within-item contrast: the same pictures were named in the same languages in the preparation and no-preparation conditions, so cross-linguistic phonological differences are held constant across the cue manipulation and cannot account for the cue benefit.

The study used a 3 (Trial Type: single, stay, switch) × 2 (Target Language: Croatian, German) × 2 (Cue Condition: preparation, no preparation) mixed design. Trial type and cue condition were manipulated within subjects, while dominant language (Croatian-dominant, German-dominant) was a between-subjects factor.

The three trial types differed in their language-switching demands. Single trials appeared in single-language blocks where the child named all pictures in one language (either Croatian or German), providing a baseline measure of naming speed without language-switching demands. Stay trials appeared in mixed-language blocks when the target language remained the same as the previous trial (e.g., German → German). Switch trials appeared in mixed-language blocks when the target language changed from the previous trial (e.g., Croatian → German).

The two cue conditions varied in the amount of preparation time available before picture presentation. In the no-preparation condition, one mixed-language block presented the language cue (flag) and picture simultaneously, providing no advance preparation time. In the preparation condition, the other mixed-language block presented the language cue 500 ms before the picture, providing time for advance language preparation.

### 2.3. Procedure

Testing took place in quiet rooms at schools, community centers, or participants’ homes. Children sat approximately 50 cm from a laptop screen and wore a headset with microphone for response recording. The experiment was programmed in PsychoPy, version 2024.2.4 (Peirce et al., 2019).

Each session began with practice trials to familiarize children with the task. The experimenter explained that they would see pictures and flags, and that the flag indicated which language to use (Croatian flag = Croatian name; German flag = German name). Children practiced naming pictures in single-language contexts before beginning the experimental blocks.

The experimental session consisted of six blocks presented in the following order. The first two blocks were single-language blocks containing 17 trials each, one in German and one in Croatian. The third block was a mixed-language block containing 51 trials, presented either with preparation time or without preparation time. The fourth and fifth blocks were again single-language blocks with 17 trials each, one in German and one in Croatian. The sixth and final block was a mixed-language block with 51 trials, presented in whichever cue condition had not been used in the third block. The order of blocks and cue conditions was counterbalanced across participants using four lists. In mixed-language blocks, the required language switched unpredictably across trials, with equal numbers of stay and switch trials. Trial sequences within each block were fixed pseudorandom orders defined by the stimulus lists, so that the counterbalanced lists varied which cue condition and block order a child received while holding sequence structure constant across children within a list. Each of the 34 pictures appeared in both single-language and mixed-language blocks, and all children completed the same practice trials before the experimental blocks.

On each trial, children saw a fixation cross for 500 ms. In the no-preparation condition, the flag and picture appeared simultaneously and children named the picture in the flagged language as quickly as possible. In the preparation condition, the flag appeared for 500 ms before the picture appeared, and children named the picture in the flagged language as quickly as possible. All sessions were audio-recorded. Speech onset times were measured from picture onset to the beginning of the child’s vocal response and extracted manually for each trial by inspecting the acoustic waveform and spectrogram of individual sound recordings in Praat, version 6.4.26 (Boersma & Weenink, 2024). The onset criterion was the earliest moment of acoustic energy attributable to the target word, identified first by listening to the recording and then by visually locating the corresponding point on the waveform and spectrogram. Trials containing hesitations or filled pauses before the correct response were retained, with onset marked at the beginning of the target-word production rather than at the hesitation, on the assumption that hesitations reflect continued lexical or phonological planning rather than a failed response. Self-corrections were rare, as the fast pace of the task left little opportunity for them; trials on which the first production was incorrect were excluded as errors rather than retained with a corrected onset time. Because German voiceless stops are aspirated whereas Croatian voiceless stops are not, the same criterion captures the aspiration phase as part of the word onset in German while capturing the burst or voicing onset in Croatian. Onset times in both languages therefore reflect the same underlying criterion—the first moment the child began producing the word—although for German words beginning with voiceless stops this may introduce a small systematic difference in absolute onset times relative to Croatian; since this applies equally across cue conditions and trial types within German, it cannot affect the critical within-language comparisons. To assess annotation reliability, approximately 10% of trials were independently annotated by a second trained annotator (inter-rater reliability: ICC = 0.998, mean absolute difference = 20.3 ms, SD = 46.9 ms, across 505 paired trials). The picture remained on screen until the child responded or 5 s elapsed.

### 2.4. Data Analysis

Speech onset times were log-transformed to reduce positive skew and better approximate normal distribution. Of the 6284 experimental trials, 1293 (20.6%) could not be assigned a speech onset time (omissions, unintelligible productions, first-production errors, or recording failures) and were excluded, leaving 4991 analyzable trials. Wrong-language productions that could nonetheless be annotated (59 trials; 1.2% of analyzable trials) were retained in the primary analyses; excluding them did not change any of the results (Section 3.5). Missingness was higher among the youngest children (33.0% of mixed-block trials for children under 7 vs. 16.7% for older children) but was closely comparable across the two cue conditions (20.8% no-preparation vs. 18.8% preparation). Accuracy and error analyses are reported in Section 3.5.

We conducted linear mixed-effects analyses using the lme4 package in R, with log-transformed speech onset time as the dependent variable. Models included random intercepts for participants and items (pictures) to account for individual differences and item-specific variation. Fixed effects were evaluated with Satterthwaite-approximated *t*-tests as implemented in the lmerTest package, and 95% confidence intervals are reported for key effects. Because random-intercept-only models can be anticonservative for within-participant manipulations, we refit each primary model with by-participant random slopes for the within-participant factors (trial type and/or cue condition); all random-slope models converged without singular fits and all conclusions were unchanged (Section 3.5). For the two theoretically important non-significant interactions (cue visibility × age and cue visibility × trial type), we additionally conducted two one-sided equivalence tests (TOST), with the smallest effect size of interest set to half the magnitude of the cue main effect.

## 3. Results

### 3.1. Descriptive Overview

**Figure 1:**
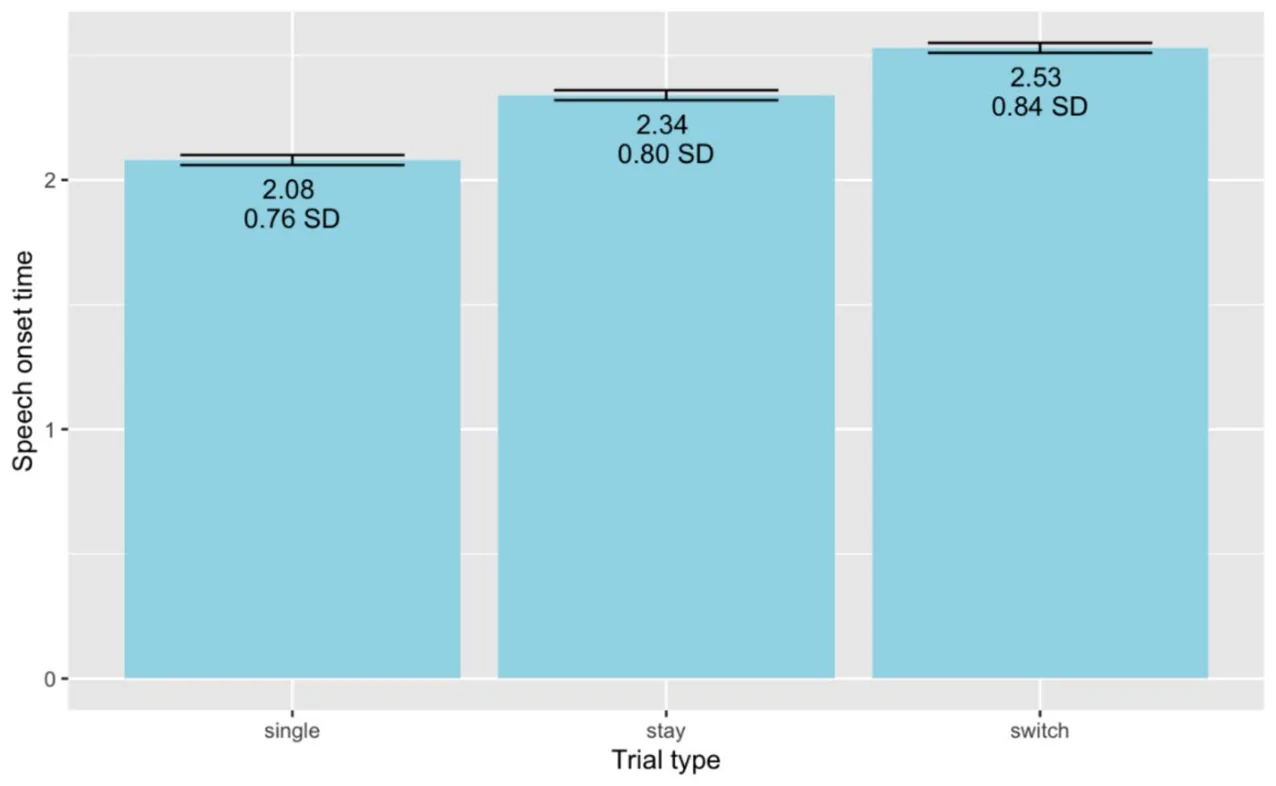
Average speech onset time (in seconds) for each trial type (single, stay, and switch). Error bars represent the standard error of the mean (SE).

Mean speech onset times increased progressively across trial types as shown in Figure 1: single trials (M = 2.08 s, SD = 0.76), stay trials (M = 2.34 s, SD = 0.80), and switch trials (M = 2.53 s, SD = 0.84). This pattern provided initial evidence for both mixing costs (stay > single) and switch costs (switch > stay). Additionally, children responded faster in the preparation condition (M = 2.25 s, SD = 0.80) than in the no-preparation condition (M = 2.64 s, SD = 0.81) within the mixed-language blocks, suggesting a substantial cue benefit.

### 3.2. Switch Costs

To examine switch costs, we analyzed only mixed-language block trials (excluding single trials), fitting a linear mixed-effects model with trial type (stay vs. switch), target language (Croatian vs. German), and dominant language (Croatian-dominant vs. German-dominant) as fixed effects, along with their interactions.

The model revealed a significant main effect of trial type (β = 0.06, SE = 0.02, 95% CI [0.02, 0.10], *p* = 0.002), confirming that children took longer to respond on switch trials compared to stay trials, pointing to a reliable switch cost. Neither the trial type × target language interaction (β = 0.02, SE = 0.03, 95% CI [−0.04, 0.09], *p* = 0.499) nor the trial type × dominant language interaction (β = 0.01, SE = 0.03, 95% CI [−0.05, 0.07], *p* = 0.716) reached significance, and the three-way interaction was also non-significant (β = −0.00, SE = 0.05, 95% CI [−0.09, 0.08], *p* = 0.919). The switch cost was unchanged with a by-participant random slope for trial type (β = 0.06, SE = 0.02, *p* = 0.004) and when continuous relative Croatian exposure replaced the binary dominance grouping (β = 0.07, SE = 0.03, *p* = 0.014).

These results provide no evidence that switch costs differed by target language or dominance group. We note, however, that the confidence intervals do not rule out small-to-moderate asymmetries; the pattern is therefore best described as an absence of evidence for asymmetry rather than positive evidence of equivalence.

### 3.3. Mixing Costs

To examine mixing costs, we compared stay trials from mixed-language blocks to single trials from single-language blocks, fitting a model with trial type (single vs. stay), target language, and dominant language as fixed effects.

The model revealed a significant main effect of trial type (β = 0.15, SE = 0.02, 95% CI [0.12, 0.19], *p* < 0.001), confirming that children took longer on stay trials in mixed blocks compared to single-language trials, pointing to a reliable mixing cost. There was also a significant interaction between trial type, target language, and dominant language (β = 0.13, SE = 0.04, 95% CI [0.04, 0.21], *p* = 0.004), which was unchanged with a by-participant random slope for trial type (β = 0.13, SE = 0.04, *p* = 0.004); a corresponding interaction emerged when continuous Croatian exposure replaced the dominance grouping (β = −0.18, SE = 0.06, *p* = 0.004).

**Figure 2:**
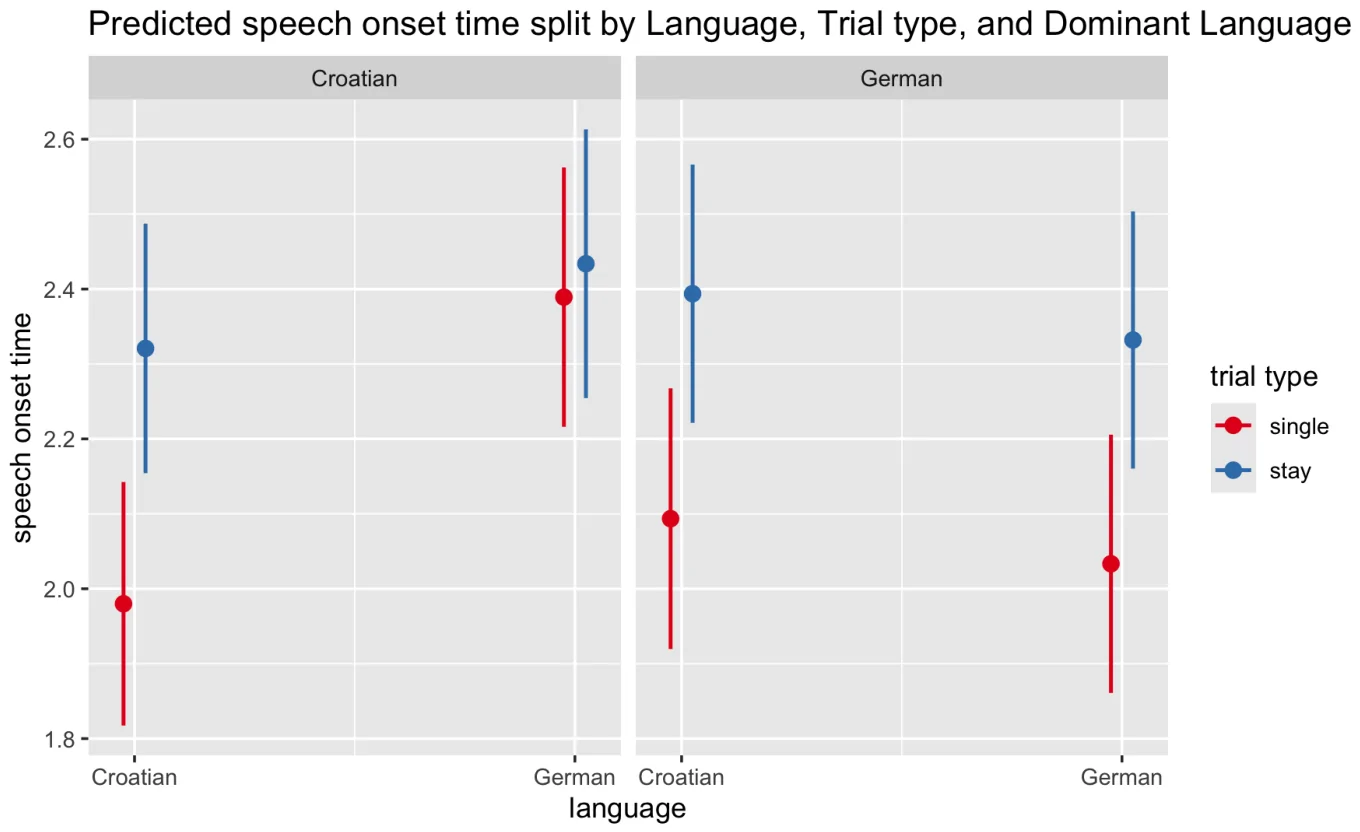
Predicted speech onset time split by Language, Trial Type (single vs. stay), and Dominant Language.

To interpret this interaction, we examined predicted values for each group as shown in Figure 2. Croatian-dominant children showed larger mixing costs in Croatian than in German, meaning that the performance difference between stay trials in the mixed block and single-language trials was greater for their dominant language. German-dominant children, by contrast, showed more comparable mixing costs across the two languages. Importantly, closer inspection of the single-trial data suggests that this interaction was at least partly driven by differences in single-trial naming latencies between the two groups: German-dominant children were faster at naming pictures in the single-language blocks than Croatian-dominant children, pointing to language-specific retrieval differences that may reflect stronger or more automatized lexical access in German compared to Croatian in this sample. This could indicate that the children’s dominant language was more robustly established in German-dominant children, leading to faster baseline retrieval rather than reflecting differences in switching-related control demands per se. Beyond this retrieval difference, the pattern of mixing costs also suggests that maintaining the dominant language in a state of readiness while simultaneously monitoring for potential language switches may impose particular cognitive load, especially in children with less frequent switching experience, as was the case for the Croatian-dominant group living in a predominantly monolingual environment.

### 3.4. Preparation Benefits

The central analysis examined whether children could utilize advance preparation time to facilitate language switching. We analyzed mixed-language block trials, fitting a model with cue visibility (preparation vs. no-preparation), trial type (stay vs. switch), age (continuous), and their interactions as fixed effects.

The model revealed a significant effect of cue visibility (β = −0.28, SE = 0.07, 95% CI [−0.41, −0.15], *p* < 0.001), indicating that children responded faster when the language cue appeared in advance, and a significant effect of age (β = −0.05, SE = 0.01, 95% CI [−0.07, −0.03], *p* < 0.001), with older children responding faster overall. The trial-type coefficient was non-significant in this model (β = 0.06, SE = 0.07, *p* = 0.365).

**Figure 3:**
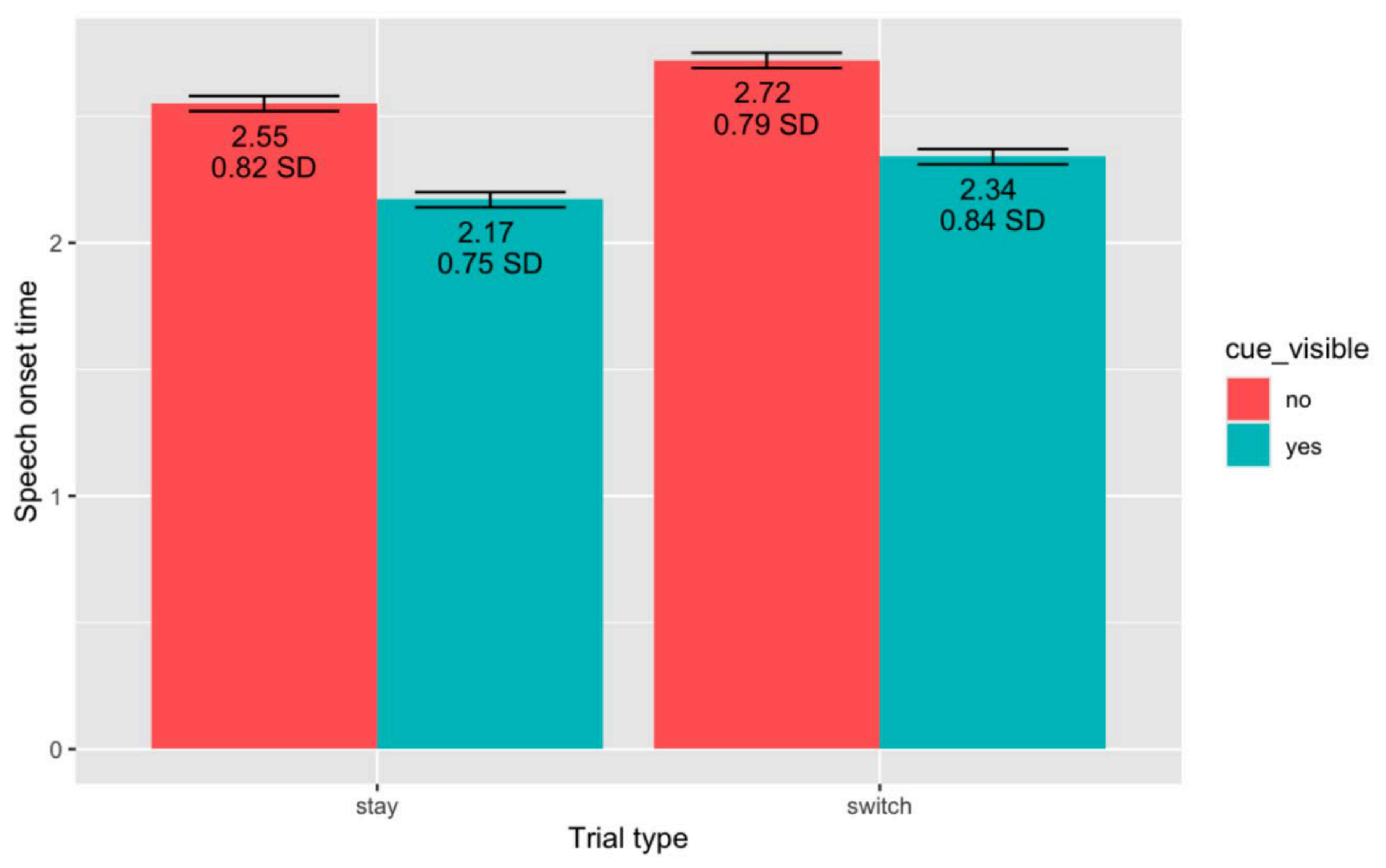
Mean speech onset times (in seconds) by trial type (stay vs. switch) and cue visibility (cue visible vs. not visible). Error bars represent ±1 standard error. Speech onset times were faster when the cue was visible, for both stay and switch trials.

Examination of the critical interactions revealed no significant moderation of the cue benefit. The cue visibility × age interaction was non-significant (β = 0.013, SE = 0.008, 95% CI [−0.002, 0.028], *p* = 0.095; *p* = 0.131 with a by-participant random slope for cue condition), and an equivalence test indicated that this interaction was significantly smaller than our smallest effect size of interest of half the cue main effect (TOST *p* < 0.001), supporting the absence of a meaningful moderation by age within this sample. The cue visibility × trial type interaction was also non-significant (β = 0.002, SE = 0.095, 95% CI [−0.18, 0.19], *p* = 0.984); for this interaction, however, the equivalence test was inconclusive (TOST *p* = 0.072), so the data do not establish that the cue benefit was equivalent for switch and stay trials—only that we found no evidence of a difference. The age × trial type interaction (β = 0.001, SE = 0.008, *p* = 0.927) and the three-way interaction (β = 0.000, SE = 0.011, *p* = 0.987) were likewise non-significant. As seen in Figure 3, children responded faster when they could anticipate the target language, to a similar degree for both switch and stay trials.

**Figure 4:**
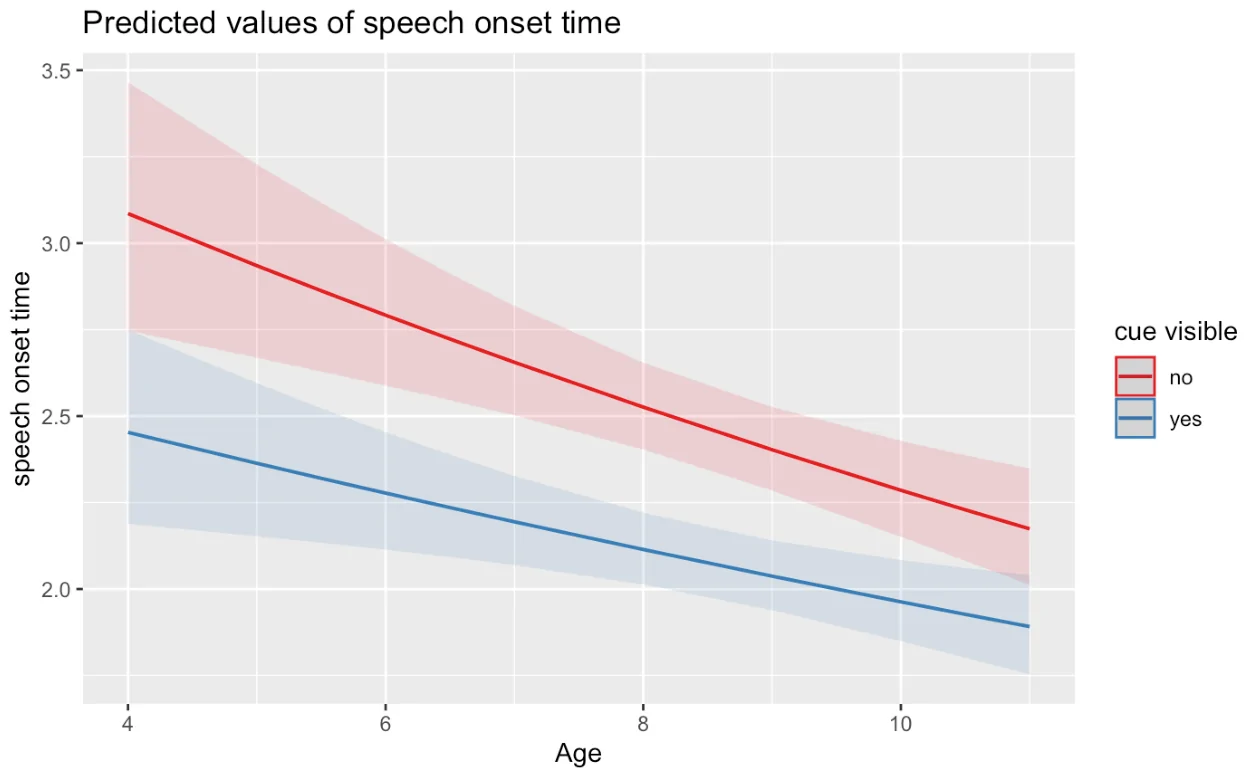
Predicted speech onset times across age, separated by cue visibility (cue visible vs. not visible).

**Figure 5:**
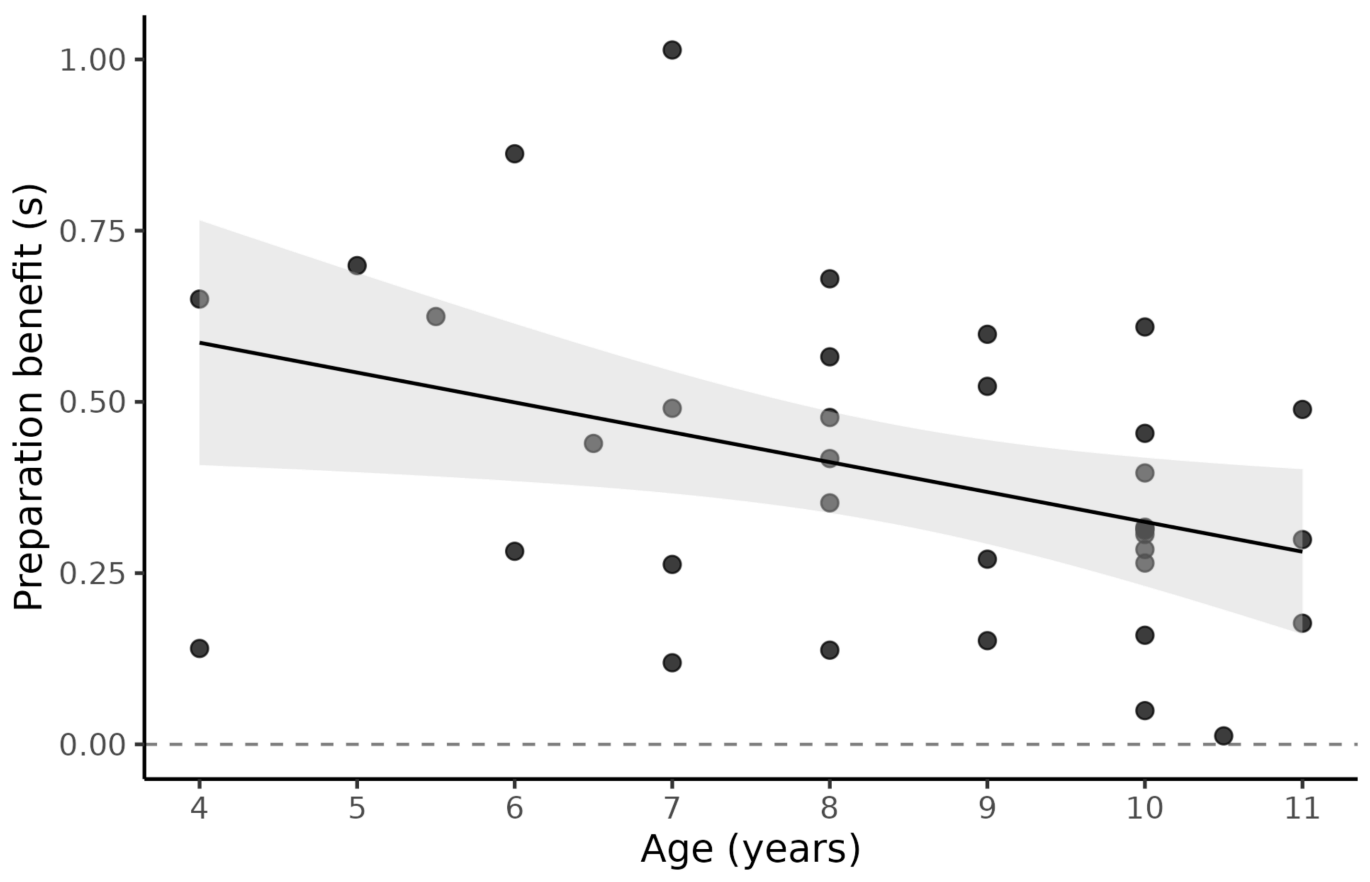
Per-participant preparation benefit (mean no-preparation RT minus mean preparation RT, in seconds) as a function of age, for the 36 children with data in both cue conditions. Every child showed a positive benefit. The solid line shows the linear fit with its 95% confidence band; the dashed line marks zero.

The absence of a significant cue visibility × age interaction is particularly noteworthy. Figure 4 displays predicted speech onset times as a function of age for both cue conditions. Both conditions show decreasing response times with age (reflecting general developmental improvements in processing speed), but critically, the gap between preparation and no-preparation conditions remains relatively constant across the age range. This pattern suggests that the youngest children in our sample benefited from advance preparation. At the individual level, every child who completed both cue conditions (36 of 36) responded faster on average in the preparation condition, and the raw preparation benefit was, if anything, numerically larger in younger children, which partly reflects their slower overall response times (Figure 5). Given the small number of children at the youngest ages, however, claims about the developmental trajectory of the benefit remain tentative.

To examine whether the youngest children in our sample could utilize advance preparation cues, we conducted a supplementary analysis focusing on the seven children under 7 years of age (ages 4.0–6.5; 469 trials in total across these seven children). A linear mixed-effects model with log-transformed speech onset times as the dependent variable, cue visibility (preparation vs. no-preparation) and trial type (stay vs. switch) as fixed effects, and random intercepts for participants and items, revealed that even these youngest children showed a highly significant preparation benefit (β = −0.20, SE = 0.03, t = −5.81, *p* < 0.001). There was also a significant main effect of trial type, with switch trials eliciting longer latencies than stay trials (β = 0.07, SE = 0.03, t = 2.08, *p* = 0.038), confirming that switching costs were present in this youngest age group. Critically, the interaction between cue visibility and trial type was non-significant (β = −0.005, SE = 0.05, t = −0.09, *p* = 0.925), indicating that the preparation benefit applied equally to both switch and stay trials. Because this analysis rests on only seven children—and thus on limited independent observations at the participant level, which the number of trials cannot compensate for—we treat it as preliminary. It nonetheless converges with the individual-level data (all seven children showed a numerically positive preparation benefit, range 0.14–0.86 s) in suggesting that the youngest children in our sample were able to make use of the advance cue.

### 3.5. Accuracy, Error Patterns, and Robustness Analyses

Accuracy analyses complement the latency results. Unanalyzable trials—overwhelmingly omissions, together with unintelligible productions and occasional recording failures—accounted for 20.6% of experimental trials, and wrong-language productions for a further 59 trials (0.9% of all trials; 1.2% of responses). Omissions were strongly concentrated in children’s weaker language: Croatian-dominant children failed to respond on 48.7% of German trials but only 3.4% of Croatian trials, while German-dominant children showed the reverse, attenuated pattern (18.6% in Croatian vs. 11.5% in German). Error rates also declined with age (35.0% for 4–6-year-olds, 20.9% for 7–8-year-olds, and 17.2% for 9–11-year-olds), consistent with the developmental pattern in latencies.

Overall accuracy did not differ meaningfully between switch (79.1%) and stay (78.5%) trials: the latency switch cost was not paralleled by a general accuracy cost, suggesting that switching taxed the time course of reconfiguration rather than its accuracy. The specific subset of wrong-language intrusions was, however, more frequent on switch (2.0%) than stay (0.8%) trials, consistent with momentary competition from the previously used language. A logistic mixed-effects model predicting whether a mixed-block trial yielded an analyzable, correct-language response (random intercepts for participants and items) showed only a significant effect of age (b = 0.21, SE = 0.07, *p* = 0.005), with older children producing more analyzable correct responses; there were no significant effects of cue condition, trial type, or their interactions (all *p* > 0.19).

Critically, there was no evidence of a speed-accuracy tradeoff across cue conditions. Accuracy was closely comparable with (79.6%) and without (78.0%) preparation, while correct-trial response times were substantially faster with preparation (2.25 s vs. 2.63 s), and this dissociation held within every age group (4–6 years: 2.62 s vs. 3.20 s, accuracy 63.4% vs. 66.3%; 7–8 years: 2.25 s vs. 2.66 s, accuracy 80.0% vs. 79.4%; 9–11 years: 2.15 s vs. 2.45 s, accuracy 85.0% vs. 81.6%). The per-child correlation between the latency benefit and the accuracy change across cue conditions was near zero (r = 0.08, *p* = 0.64). The preparation benefit therefore reflects genuine facilitation of response planning rather than a shift along a speed-accuracy continuum—including for the youngest children, whose benefit of roughly 0.6 s was accompanied by no meaningful accuracy difference between conditions.

The main conclusions were also robust across model specifications. Excluding the 59 wrong-language trials left the cue benefit essentially unchanged (β = −0.28, SE = 0.07, *p* < 0.001). Refitting the primary models with by-participant random slopes likewise left all conclusions intact (cue benefit: β = −0.28, SE = 0.07, *p* < 0.001; switch cost: β = 0.06, SE = 0.02, *p* = 0.004; mixing-cost three-way interaction: β = 0.13, SE = 0.04, *p* = 0.004), and replacing the binary dominance grouping with continuous relative Croatian exposure reproduced the switch-cost and mixing-cost patterns (Section 3.2 and Section 3.3).

## 4. Discussion

The most important finding of this study is that even young bilingual children (4–6 years) showed substantial and consistent benefits from advance preparation time during language switching. When children saw the language cue 500 ms before the picture appeared, they responded significantly faster than when the cue and picture appeared simultaneously. Critically, this preparation benefit did not vary significantly with age across the 4–11 year range studied—and it was present in every child with data in both conditions—potentially suggesting that the capacity to use advance language cues emerges early in bilingual development. However, the sample was small and unevenly distributed across ages (only two 4-year-olds and two 5-year-olds), so although developmental stability is supported by an equivalence test against a pre-specified smallest effect of interest (Section 3.4), it warrants replication in larger samples. Second, because the advance cue also acts as a temporal warning signal, the benefit cannot be unambiguously attributed to language-specific proactive control as opposed to more general preparatory processes such as alerting or temporal orienting. We therefore describe our findings in terms of cue-based preparation and treat their language-specificity as an open question.

Regarding the second caveat, several features of the design and results make a pure alerting or foreperiod account unlikely to explain the full benefit, although they do not rule it out. The same flag cue was presented in both conditions, with only its temporal separation from the picture differing; the cue is semantically language-specific and must be decoded against culturally acquired knowledge rather than merely detected; and it specifies task content—which language to speak—rather than response timing. Moreover, the benefit was large: roughly 390 ms in raw response times on correct trials (2.63 s vs. 2.25 s), which is an order of magnitude greater than canonical alerting and foreperiod effects (typically tens of milliseconds), although such effects can scale with children’s slower overall response times.

This finding adds to prevailing accounts of cognitive control development, which emphasize the gradual emergence of proactive control across childhood (Chatham et al., 2009; Lucenet & Blaye, 2014; Munakata et al., 2012). Studies using classic cognitive control tasks (such as the AX-Continuous Performance Task or cued task-switching paradigms) have consistently found that children under 7–8 years show minimal evidence of proactive preparation, relying instead on reactive strategies to resolve conflict after it arises. Our results are consistent with the possibility that, in the domain of bilingual language control, anticipatory mechanisms can be engaged earlier than such accounts would predict.

Bilingual children’s daily lives provide extensive practice at selecting a language from contextual cues (e.g., “we speak German with Oma”). This everyday reliance on contextual cues aligns with the Adaptive Control Hypothesis (Green & Abutalebi, 2013), which proposes that the cognitive demands of language control are inherently contextual and shaped by the communicative situations bilinguals regularly encounter. In dual language contexts, for instance, a salient environmental cue (such as the arrival of a new addressee with whom the speaker habitually uses their other language) may trigger an anticipatory switch before any linguistic interference has even arisen. Everyday experience of this kind gives bilingual children extensive practice at using contextual cues to prepare an upcoming language, and this cue-driven preparation may be precisely what our flag-based manipulation taps into. We emphasize, however, that cue detection itself is not what our manipulation measures: the flag was presented, and detected, in both conditions, and what differed was whether its temporal separation from the picture allowed the detected cue to be used for advance preactivation. The present data thus license the claim that children used the advance interval to initiate preparatory processing, while the precise content of that preparation remains to be determined.

The first explanation is that linguistic preparation imposes lower working-memory demands than proactive control in classic cognitive tasks. Proactive control in many cognitive tasks requires maintaining abstract rules or stimulus-response mappings in working memory; a capacity that develops gradually across childhood (Chevalier et al., 2014; Gonthier et al., 2019; Troller-Renfree et al., 2020). In contrast, language preparation may rely on more automatic, less working-memory-intensive processes. When children see the Croatian flag, they may automatically pre-activate their Croatian lexicon through spreading activation, without needing to actively maintain abstract task rules. This automaticity could allow proactive language preparation to emerge earlier than proactive control in tasks requiring deliberate rule maintenance.

The second explanation is that the salient, meaningful cues used in language-switching tasks are particularly effective at eliciting anticipatory preparation in young children. The language cues in our task (national flags representing Croatian and German) were highly salient, meaningful, and strongly associated with the target languages through children’s cultural knowledge. Such rich, semantically meaningful cues may be particularly effective at eliciting proactive preparation in young children, compared to the arbitrary cues often used in cognitive control tasks. This account aligns with research by Chevalier et al. (2015), who demonstrated that young children can engage proactive control when task conditions provide sufficiently salient and informative cues. In their study, 5-year-olds who predominantly relied on reactive control in standard cued task-switching paradigms were able to engage proactive preparation when the cue was made more salient by disappearing before the target appeared, forcing children to use the cue proactively rather than relying on it reactively. This suggests that young children may have latent proactive capabilities that are revealed only when tasks provide optimal cuing conditions, i.e., conditions that our culturally meaningful flag cues may have provided.

Regardless of which explanation(s) prove most accurate, our findings indicate that young bilingual children can use advance language cues to speed their responses during language switching. This capability has important implications for understanding both bilingual language control and cognitive development more broadly.

The substantial preparation benefit raises questions about the specific processes engaged during the cue-picture interval. What, precisely, are children doing with this advance information? Several mechanisms are plausible, and understanding these processes is crucial for developing a comprehensive model of proactive language control in childhood. To ground this question, it is useful to consider what models of speech production say about the stages at which preparation could operate. In the WEAVER++ model (Levelt et al., 1999), word production proceeds from lexical selection through morphological and phonological encoding, where phonemic segments are retrieved and assembled, before phonetic encoding and articulation begin. Critically, phonological encoding in bilingual speakers is thought to involve language-specific condition-action rules that govern what is done with activated phonological information depending on the target language (Roelofs & Verhoef, 2006). On this view, a language cue such as a national flag could in principle trigger language-specific phonological routines before the picture appears, priming not just a general readiness to speak but the phonological properties characteristic of the cued language: for German, this might include preparing for final obstruent devoicing or particular vowel quality distinctions; for Croatian, activating characteristic consonant cluster patterns or stress assignment rules. The preparation benefit we observed is consistent with this kind of early phonological tuning: by the time the picture appeared, children may already have begun to configure their phonological system for the target language, reducing the encoding work required at picture onset.

Another possibility is that children use the cue to pre-activate the target language lexicon before the picture appears. This pre-activation could involve increasing baseline activation levels across the target-language lexicon, making those words more accessible for subsequent selection (Verhoef et al., 2009). According to spreading activation models (Collins & Loftus, 1975), when children see the Croatian flag, this could trigger automatic activation of Croatian phonological and lexical representations, raising their baseline activation levels. This increased readiness would then facilitate lexical selection when the picture appears, reducing the time needed to access the appropriate word form.

Such spreading activation processes could explain why preparation benefited both stay and switch trials similarly; in both cases, seeing the cue allows the relevant language network to be strengthened. On stay trials, the cue reinforces the already-active language network, perhaps counteracting any decay that might have occurred since the previous trial. On switch trials, the cue pre-activates the new target language, reducing the activation difference between languages and facilitating the switch. The absence of a detectable difference in the benefit across trial types is consistent with preparation operating primarily through activation mechanisms rather than through processes specifically targeted at switching, although the statistical equivalence of the benefit across trial types was not established (Section 3.4), so this interpretation remains tentative.

Alternatively or additionally, children might use the cue to pre-inhibit the non-target language proactively. If the German flag appears, children could begin inhibiting Croatian words before the picture is even presented. According to the Inhibitory Control Model (Green, 1998), language control involves not just activating the target language but also actively suppressing the non-target language to prevent interference. However, several aspects of our data seem less consistent with a strong pre-inhibition account. First, preparation did not selectively reduce switch costs, where pre-inhibition of the previously active language should be most beneficial. If children were using the preparation interval primarily to inhibit the non-target language, we would expect switch trials to show disproportionately large preparation benefits, as the preparation time would allow completion of the inhibitory process before the picture appears. The absence of such a selective benefit suggests that pre-inhibition may not be the primary mechanism engaged during preparation. Converging evidence comes from Declerck et al. (2021), who found that a longer cue-to-stimulus interval did not reduce the cognate facilitation effect in bilingual picture naming, arguing against the view that preparation operates primarily through suppression of the non-target language’s phonological representations.

Children might also use the preparation interval to engage meta-cognitive strategies, such as mentally rehearsing the target language (“Okay, it’s German this time”) or recalling recent examples of words in that language. Such strategies would require more deliberate, controlled processing but could become increasingly automatic with experience. For instance, upon seeing the German flag, a child might engage in inner speech in German, perhaps recalling the German word used on the previous trial or mentally preparing common German responses they expect might be relevant. These meta-cognitive strategies could be particularly important for younger children or children with less balanced bilingualism, who may need to engage more deliberate control processes to manage language selection. Research on the development of meta-cognitive monitoring suggests that children’s ability to regulate their own cognitive processes improves substantially across middle childhood (Flavell, 1979; Schneider, 2010), and there is evidence that meta-cognitive awareness of language use in particular emerges relatively early in bilingual children who must routinely reflect on which language is appropriate in a given context (Bialystok, 2001). With age and experience, such initially effortful strategies might become more automatic, allowing children to prepare language responses more efficiently; a process consistent with the broader shift from controlled to automatic processing described in skill acquisition frameworks (Anderson, 1982; Shiffrin & Schneider, 1977). However, if meta-cognitive strategies were the primary mechanism driving preparation benefits, we might expect developmental increases in those benefits as children’s meta-cognitive abilities mature, a pattern we did not observe. The developmental stability of preparation benefits across the 4–11 year age range is more consistent with accounts in which proactive control operates through relatively automatic mechanisms that do not depend heavily on deliberate reflection. This suggests that either (a) even young children can engage effective meta-cognitive strategies in this linguistically familiar context, given their extensive real-world experience with cue-based language selection, or (b) more automatic mechanisms such as spreading lexical activation, proactive inhibition of the non-target language, or task-set reconfiguration are primarily responsible for the preparation benefit, with meta-cognitive strategies playing a secondary or supplementary role.

Finally, although the stability of the preparation benefit across the 4–11 year range might seem to imply that all children were doing the same thing during the cue-picture interval, equivalent response-time reductions need not reflect equivalent depth of phonological encoding. Cross-linguistic differences in bilingual children’s phonological processing operate at multiple levels—individual sound categories, phonotactic constraints, and suprasegmental features—and early phonetic processing is shaped substantially by children’s language environment and relative exposure (McCarthy & Skoruppa, 2023). Younger or less proficient children may have been activating the target-language phonological system in a relatively coarse-grained way, bringing online a broad readiness to produce sounds in that language, whereas older and more proficient children may have been encoding finer-grained properties such as specific stress patterns or language-specific segmental contrasts. Acoustic analyses of children’s speech output—for example, voice onset time or vowel formants as a function of preparation condition—and ERP paradigms with cognate manipulations, similar to those used with adults (Declerck et al., 2021; Verhoef et al., 2009), would be well positioned to test this possibility.

In contrast to predictions of the Inhibitory Control Model (Green, 1998), we found symmetrical switch costs: children showed similar switching costs regardless of whether they were switching into or out of their dominant language. According to Green’s model, returning to the dominant language should incur larger switch costs because stronger inhibition was required to suppress it while using the weaker language. The absence of this asymmetry in our child sample suggests several possibilities. Children may employ less language-specific inhibition than adults, relying instead on domain-general selection mechanisms that treat both languages more equally. This possibility aligns with proposals that inhibitory control mechanisms in bilingualism may strengthen with age and expertise (Bialystok & Viswanathan, 2009).

Additionally, the concept of a stable “dominant” language may be less applicable to bilingual children than to adults. While our questionnaire data confirmed group-level differences in language exposure and use, individual children may experience considerable variability in dominance across contexts and over developmental time (Kubota et al., 2020). Such fluidity could reduce the systematic inhibitory differences between languages that produce asymmetrical switch costs in adults. The symmetrical pattern we observed aligns with some prior child studies (Declerck et al., 2015) but contrasts with others reporting asymmetries (Gross & Kaushanskaya, 2015). These mixed findings suggest that switch cost asymmetries in children may be more variable and context-dependent than in adults, potentially depending on factors such as the specific language pairs, children’s proficiency profiles, and task demands.

While switch costs were symmetrical and unaffected by dominance, mixing costs showed a more nuanced pattern. Croatian-dominant children showed larger mixing costs when speaking Croatian compared to German, while German-dominant children showed more balanced costs across languages. This interaction suggests that mixing costs (which reflect the sustained demands of maintaining dual-language readiness) are more sensitive to language dominance and exposure patterns than are transient switch costs.

However, closer examination reveals that this interaction was at least partially driven by differences in baseline naming performance rather than exclusively by differences in sustained control demands. Croatian-dominant children showed relatively slow naming latencies in German during single-language blocks, which meant that the difference between single-language and mixed-language German trials (i.e., the mixing cost) was comparatively small. In other words, their German was already slow in the “easier” single-language context, leaving less room for additional slowing in the mixed context. In contrast, their Croatian single-trial performance was fast, creating a larger difference when Croatian was embedded in a mixed-language block. This pattern suggests that the observed interaction reflects, at least in part, language-specific retrieval differences (possibly weaker lexical access or less automatized naming in the non-dominant German) rather than purely differences in how control is applied across languages.

That said, beyond these retrieval differences, the pattern is also consistent with genuine differences in sustained control demands. For Croatian-dominant children living in Croatia, Croatian is the default, highly accessible language used in most daily contexts. Maintaining Croatian in a mixed-language context (where it must be held in check and monitored to prevent unwanted intrusions) may require particular effort because it must be suppressed below its typical high baseline. In contrast, these children’s experience with German is more limited and often occurs in contexts already requiring language selection (e.g., speaking with bilingual family members), making the mixed-language task demands less discrepant from their typical German-use contexts. This interpretation aligns with findings from Costa and Santesteban (2004), who proposed that mixing costs reflect the effort of maintaining languages at activation levels different from their habitual states. However, our child data add complexity: the interaction between language dominance and mixing costs appears to be multiply determined, reflecting both language-specific retrieval efficiency and the sustained monitoring demands of dual-language contexts. Future research should carefully distinguish these components, for instance by including independent measures of lexical retrieval speed to partial out baseline naming differences from control-related costs. An additional and important caveat is that language dominance was confounded with sociolinguistic environment in this design: the Croatian-dominant children lived in Croatia and the German-dominant children lived in Austria, so the two groups differed not only in dominance but also in country, community language, schooling, and everyday switching demands. Group differences reported here therefore index this bundle of factors rather than dominance in isolation, and disentangling them would require, for example, comparing children with the same dominance profile across different community-language contexts.

### Limitations and Future Research

Several limitations should be acknowledged, two of which bear directly on the central claims. First, the design included no neutral or non-informative cue condition: the advance cue was both informative about the target language and a temporal warning signal. Faster responses after an advance cue are therefore compatible with language-general preparatory processes—alerting, temporal orienting, or generic response readiness—as well as with language-specific preparation. We note that the absence of a neutral-cue condition reflects the prevailing methodological standard in both the developmental proactive-control literature (Chatham et al., 2009; Chevalier et al., 2015; Lucenet & Blaye, 2014) and the bilingual language-switching literature (Fink & Goldrick, 2015; Mosca & Clahsen, 2016; Verhoef et al., 2009; Liu & Chaouch-Orozco, 2025), and the considerations outlined above make a pure alerting account unlikely to explain the full benefit; they do not, however, constitute a rigorous test. Future studies should include a neutral-cue baseline (e.g., an uninformative symbol presented at the same advance interval) to isolate the language-specific component, and studies using neural measures or manipulating the informational content of cues could further address this question. Second, our preparation interval was fixed at 500 ms. A single interval cannot reveal the time course of preparation: 500 ms may be long enough for even young children to complete some preparatory processing, while being too short to expose qualitative age differences in how preparation unfolds. A parametric manipulation of the cue-stimulus interval (e.g., 200, 500, and 1000 ms) would provide stronger evidence about developmental change in preparation, including the possibility that anticipatory activation dissipates at longer intervals (Liu & Chaouch-Orozco, 2025). Third, our sample included children with varying degrees of bilingual proficiency and language exposure. While we categorized children as Croatian-dominant or German-dominant based on questionnaire data, more fine-grained assessment of proficiency and exposure patterns might reveal additional nuances in how language experience shapes control mechanisms. Relatedly, the sample was modest in size and unevenly distributed across age, with very few children at the youngest ages; although the preparation benefit was observed in every child and survived an equivalence test for moderation by age, estimates involving age and the youngest subgroup should be treated as preliminary.

## 5. Conclusions

This study provides some of the first evidence that young bilingual children, including those as young as 4–6 years, can make use of advance preparation cues during language switching: children responded faster when the language cue preceded the picture, the benefit was observed in every child with data in both cue conditions, and it did not differ detectably across the sampled age range. Our findings are consistent with the possibility that bilingual experience supports the early use of anticipatory processes in the language domain, but this interpretation awaits confirmation with larger samples and designs that can isolate language-specific preparation. Beyond illuminating proactive control, this study revealed important dissociations between transient and sustained control processes in bilingual language switching. While switch costs did not differ detectably by target language or dominance group, mixing costs showed sensitivity to both group and target language—a pattern that partly reflects baseline differences in naming speed and should therefore be interpreted with caution.

These findings contribute to the understanding of bilingual language control in children and suggest that bilingual children can use contextual information to support language selection from early on in development. The work opens up new avenues for research on the neural bases of early language control, the role of bilingual experience in shaping cognitive development, and the design of optimal environments to support bilingual children’s language learning and use.

## Figures and Tables

**Table 1:** Summary of Croatian and German exposure between two child bilingual groups.

Variable	Croatian-Dominant		German-Dominant	
	M	SD	M	SD
Age				
Age of acquisition (Croatian)	0.00	0.00	0.08	0.34
Age of acquisition (German)	4.50	2.61	0.08	0.34
Language use—Croatian				
Friends	0.99	0.04	0.31	0.14
Home	0.97	0.06	0.50	0.20
Media	0.85	0.09	0.18	0.19
School	0.95	0.04	0.00	0.00
Language use—German				
Friends	0.02	0.04	0.72	0.14
Home	0.03	0.06	0.50	0.20
Media	0.15	0.09	0.82	0.19
School	0.05	0.04	1.00	0.00
Language proficiency				
Croatian	14.00	1.75	12.26	2.18
German	10.15	1.42	13.79	1.78

## Data Availability

The data and the analysis script is available on: https://osf.io/y7a3e/overview?view_only=28475ed972f84bc983a4f45edf1de019, accessed on 2 March 2026.
